# Hypotensive and Angiotensin-Converting Enzyme Inhibitory Activities of *Eisenia fetida* Extract in Spontaneously Hypertensive Rats

**DOI:** 10.1155/2015/349721

**Published:** 2015-12-22

**Authors:** Shumei Mao, Chengde Li

**Affiliations:** Department of Pharmacology, Key Laboratory of Applied Pharmacology of Shandong Province, Weifang Medical University, Weifang, Shandong 261053, China

## Abstract

*Objectives*. This study aimed to investigate the antihypertensive effects of an* Eisenia fetida* extract (EFE) and its possible mechanisms in spontaneously hypertensive rats (SHR rats).* Methods*. Sixteen-week-old SHR rats and Wistar-Kyoto rats (WKY rats) were used in this study. Rats were, respectively, given EFE (EFE group), captopril (captopril group), or phosphate-buffered saline (PBS) (normal control group and SHR group) for 4 weeks. ACE inhibitory activity of EFE* in vitro* was determined. The systolic blood pressure (SBP) and diastolic blood pressure (DBP) were measured using a Rat Tail-Cuff Blood Pressure System. Levels of angiotensin II (Ang II), aldosterone (Ald), and 6-keto-prostaglandin F1 alpha (6-keto-PGF_1*α*_) in plasma were determined by radioimmunoassay, and serum nitric oxide (NO) concentration was measured by Griess reagent systems.* Results*. EFE had marked ACE inhibitory activity* in vitro* (IC_50_ = 2.5 mg/mL). After the 4-week drug management, SHR rats in EFE group and in captopril group had lower SBP and DBP, lower levels of Ang II and Ald, and higher levels of 6-keto-PGF_1*α*_ and NO than the SHR rats in SHR group.* Conclusion*. These results indicate that EFE has hypotensive effects in SHR rats and its effects might be associated with its ACE inhibitory activity.

## 1. Introduction

Hypertension, an age related chronic disease affecting millions of people worldwide, is a crucial risk factor for myocardial infarction, heart failure, stroke, and renal damage [1, 2]. The agents used to treat hypertension in clinic include diuretics, calcium channel blockers, beta adrenergic antagonists, angiotensin-converting enzyme (ACE) inhibitors, and angiotensin II type I receptor antagonists [3]. Besides the hypotensive effects, some of these agents, such as ACE inhibitors, have beneficial effects on hypertensive damage of cardiovascular organs including blood vessels, kidney, and heart [4, 5].

The renin-angiotensin system (RAS), both the circulating RAS and renal RAS, plays an important role in the development and progress of hypertension and hypertensive damage of cardiovascular organs [6]. Renin, a substance released mainly from the kidney, cleaves angiotensinogen at the N-terminus to form Ang I [7, 8]. Ang I is rapidly converted into Ang II by angiotensin-converting enzyme (ACE) which is located on endothelial cells and on membranes of various other cells [9]. Although there are several bioactive angiotensin peptides, such as Ang I, Ang II, Ang III, Ang IV, and Ang 1–7, Ang II is the major effector of RAS system. Ang II can constrict vascular smooth muscle, promote aldosterone production, stimulate catecholamines release, regulate sodium transport in kidney, and exacerbate the remodeling of cardiovascular organs [10, 11]. It has been shown that inhibition of the production of Ang II by ACE inhibitors can produce strong hypotensive effects and protective effects on cardiovascular organs [4, 12]. Recently, efforts have been made to find new drugs that possess ACE inhibitory activity [13–15].

Extracting biologically active compounds from traditional herbs is one of the methods obtaining new active drugs. Some traditional Chinese drugs are believed to have beneficial effects on cardiovascular disease and some of them are used to treat hypertension in clinic [16–19]. Antihypertensive agents, decreasing blood pressure via various mechanisms, have recently been isolated from many traditional herb drugs [20–22], and some of them have been shown to have ACE inhibitory activity [23].

The clinical application of traditional medicine earthworm such as* Lumbricus rubellus*,* Eisenia fetida*, and* Lumbricus bimastus* and compounds extracted from such earthworm species has been well documented. Such species and their extracted compounds have been reported to have various pharmacological effects including cardiovascular protection [24, 31–34]. For example, Lai et al. [25] reported that lumbrokinase extracted from earthworm ameliorated second-hand smoke-induced cardiac fibrosis; Lee et al. [26] found SPP-501, a novel proteinase fraction purified from the earthworm, showed both antithrombotic and fibrinolytic activities when orally administered in venous thrombosis model rats. However, less is known about the effects of earthworm and its extracts on blood pressure and RAS system. Thus, this study was undertaken to investigate the effects on blood pressure and RAS system in SHR rats.

## 2. Materials and Methods

### 2.1. Animals

Sixteen-week-old male SHR rats and WKY rats (weighing 180–210 g) were purchased from Shanghai SLAC Laboratory Animal Company (China). Every 5 rats were housed in standard cages with controlled temperature (25 ± 2°C) and a 12 : 12 h light/dark cycle. The rats were fed a regular chow diet and provided with free access to food and water. The experiments were conducted strictly in accordance with the national guidelines for the care and use of laboratory animals. All the protocols involving animals in the study were approved by the Committee on the Ethics of Animal Experiments of Weifang Medical University and efforts were made to minimize the animal's suffering. Twenty-seven SHR rats were randomly divided into SHR group, EFE group, and captopril group (9 rats in each group); 9 WKY rats served as normal control group.

### 2.2. Preparation of Extract

We obtained an extract from* Eisenia fetida*, a species of earthworm, by gel-filteration chromatography. Briefly,* Eisenia fetida* was washed and homogenized in purified water. The* Eisenia fetida* homogenate was centrifuged, and the supernatant was collected and stored at minus 80°C for further purification. EFE was extracted from the supernatant by gel-filtration chromatography (Sephadex G-50) using Amersham ÄKTA Purifier 100 (Amersham Company, Sweden), freeze-dried using a freeze-dry machine under vacuum at minus 45°C, and stored at minus 20°C.

### 2.3. Evaluation of ACE Inhibitory Activity* In Vitro*

ACE inhibitory activity of EFE* in vitro* was determined according to previously described methods of Cushman and Cheung [27] modified by Sato et al. [28]. Briefly, 20 *μ*L sample solution was mixed with 150 *μ*L Hippuryl-His-Leu (7.6 mM) in 50 mM sodium borate buffer. Fifty microliters of ACE solution (25 mU/mL, dissolved in 50 mM sodium borate buffer) was added to the above mixed solution. Then 250 *μ*L 1 M HCl was added to the mixture to stop the reaction after the incubation at 37°C for 30 min. Subsequently, 2 mL of ethyl acetate was added to extract hippuric acid. After centrifugation at 3000 rpm for 15 min, 0.5 mL supernatant was collected into a glass tube and dried for 2 h at 30°C. The absorbance of the freshly extracted hippuric acid (redissolved in 3 mL distilled water) was measured at 228 nm with a UV spectrophotometer (Shimadzu, Japan) and the ACE inhibitory activity was calculated.

### 2.4. Drug Treatment

The freshly prepared EFE solution (dissolved in PBS) was daily given to the SHR rats in EFE group by intraperitoneal injection at the dose of 400 mg/kg body weight for 4 weeks. SHR rats in captopril group were daily given captopril (50 mg/kg body weight, dissolved in PBS) by intragastric administration for 4 weeks. The SHR rats in the SHR group and WKY rats only received intraperitoneal injection of PBS.

### 2.5. Blood Pressure Measurement

Before and after the 4 weeks of treatment, SBP and DBP of the animals were measured by a tail-cuff technique using a noninvasive blood pressure measurement system (Chengdu TME Technology Company, China). Before the measurement, rats were kept in a warm box (37°C) for 5 min. SBP and DBP were determined 3 times blind to the randomization sequence on each time point and the mean values were used as the result.

### 2.6. Immunoradiometric Assay of Ang II, Ald, and 6-Keto-PGF_1*α*_

Rats were anesthetized and blood samples were collected from the left carotid artery. Blood samples for determinations of circulating Ang II, Ald, and 6-keto-PGF_1*α*_ were collected into tubes containing disodium EDTA and protease inhibitors; subsequently samples were centrifuged at 3000 rpm for 10 min at 4°C and the collected plasma was stored at minus 20°C for further determinations. Concentrations of circulating Ang II, Ald, and 6-keto-PGF_1*α*_ were measured by commercial radioimmunoassay kits (Beijing North Institute of Biological Technology Company, China) following the company's protocol.

### 2.7. NO Assay

Blood samples for determinations of serum NO concentrations were collected into tubes without disodium EDTA and centrifuged at 3000 rpm for 10 min at 4°C. Serum NO determination was measured using Griess reagent systems, as previously described [29].

### 2.8. Statistical Analysis

Data of SBP and DBP and concentrations of circulating Ang II, Ald, 6-keto-PGF_1*α*_, and NO were expressed as mean ± SD and analyzed using SPSS 13.0. A one-way analysis of variance (ANOVA) followed by Student-Newman-Keuls (SNK) test was used to examine differences between groups. *P* value less than 0.05 was used as a criterion for statistical significance.

## 3. Results

### 3.1. ACE Inhibitory Activity of the Earthworm Extract

ACE inhibitory activity was expressed as IC_50_. The IC_50_ value was defined as the concentration of inhibitor required to inhibit 50% of ACE activity under the assayed conditions. In this study, the extract from earthworm has an IC_50_ value of 2.5 mg/mL, which meant it had a certain degree of ACE inhibitory activity* in vitro*.

### 3.2. Antihypertensive Effects

**Figure 1:**
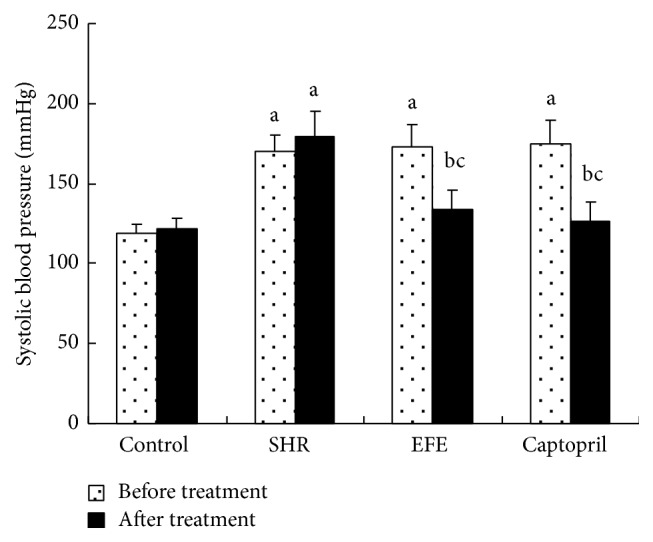
Systolic blood pressure of the rats. Each bar is the mean ± SD of the systolic blood pressure of each group. Differences between groups were determined by ANOVA procedure and S-N-K test. ^a^ *P* < 0.05 versus the control group, ^b^ *P* < 0.05 versus the SHR group, and ^c^ *P* < 0.05 versus the basal blood levels.

**Figure 2:**
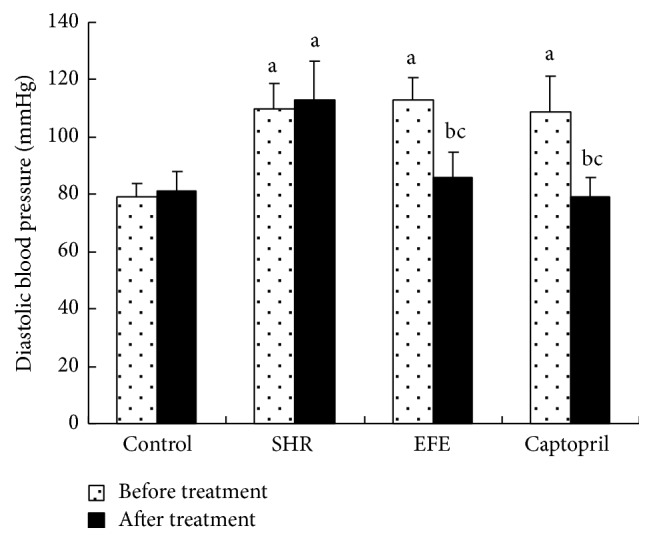
Diastolic blood pressure of the rats. Each bar is the mean ± SD of the diastolic blood pressure of each group. Differences between groups were determined by ANOVA procedure and S-N-K test. ^a^ *P* < 0.05 versus the control group, ^b^ *P* < 0.05 versus the SHR group, and ^c^ *P* < 0.05 versus the basal blood levels.

In order to investigate the effects of chronic EFE administration on blood pressure in SHR rats, we measured the basal SBP and DBP before the initiation of the drug treatment and measured them again after the 4 weeks of treatment. Prior to the drug treatment, SHR rats in SHR group, EFE group, and captopril group had much higher basal SBP and DBP than the WKY rats in the control group (all *P* < 0.05), while no marked differences in SBP and DBP were found between the 3 groups of SHR rats (all *P* > 0.05). Following the 4 weeks of treatment, SHR rats in SHR group and WKY rats in the control group treated with PBS showed no marked reduction of SBP and DBP if compared to their basal blood pressure (all *P* > 0.05); however, SHR rats in the EFE group and captopril group showed significant lower SBP and DBP than those of the SHR group and their respective basal levels (all *P* < 0.05); moreover, their SBP and DBP were similar to those of the WKY rats in the control group (all *P* > 0.05) (shown in Figures 1 and 2).

### 3.3. Concentrations of Circulating Ang II and Ald Response to Treatment

**Figure 3:**
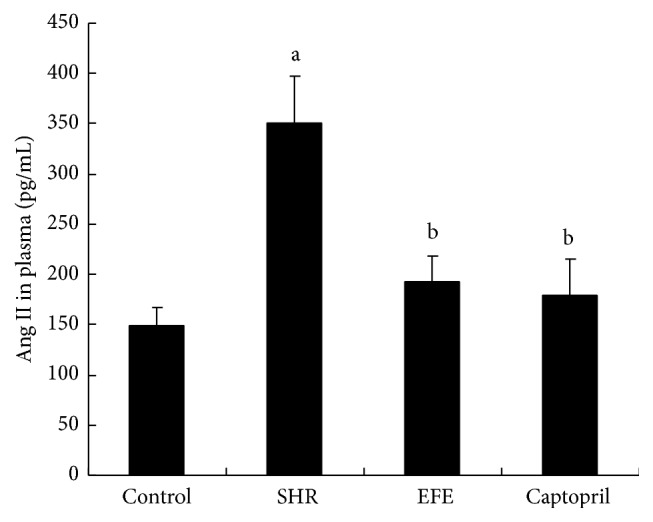
Concentrations of Ang II in plasma rats. Each bar is the mean ± SD of concentration of circulating Ang II in each group. Differences between groups were determined by ANOVA procedure and S-N-K test. ^a^ *P* < 0.05 versus the control group, ^b^ *P* < 0.05 versus the SHR group.

**Figure 4:**
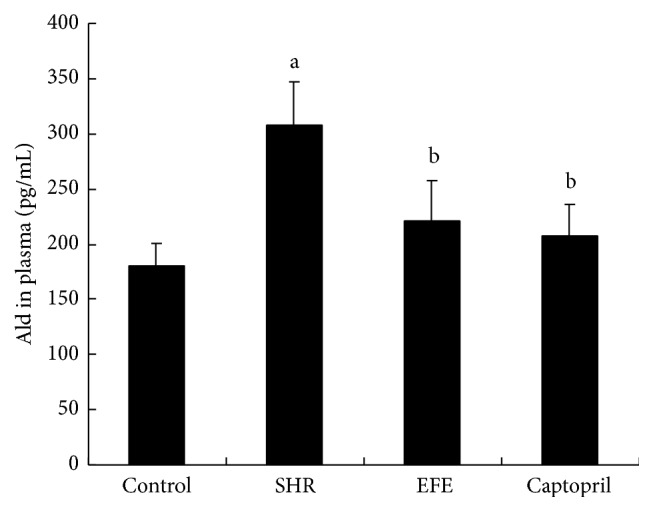
Concentrations of Ald in plasma rats. Each bar is the mean ± SD of concentration of circulating Ald in each group. Differences between groups were determined by ANOVA procedure and S-N-K test. ^a^ *P* < 0.05 versus the control group, ^b^ *P* < 0.05 versus the SHR group.

Subsequent to the 4 weeks of treatment, concentrations of circulating Ang II and Ald were measured by radioimmunoassay. As shown in Figures 3 and 4, compared to WKY rats in the control group, PBS treated SHR rats in SHR group showed markedly higher levels in these two parameters (both *P* < 0.05). EFE treated SHR rats had markedly lower levels of Ang II and Ald than the PBS treated SHR rats (*P* < 0.05) and their levels of these two parameters were similar to those of the WKY rats and the captopril treated SHR rats (all *P* > 0.05).

### 3.4. Concentrations of Circulating 6-Keto-PGF_1*α*_ and NO Response to Treatment

**Figure 5:**
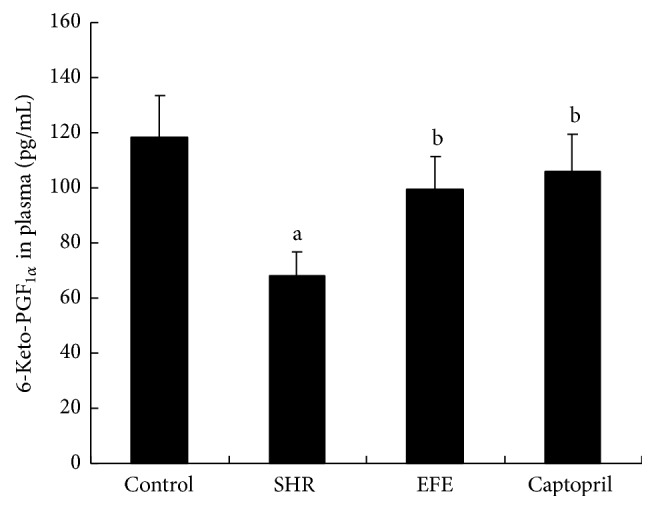
Concentrations of 6-keto-PGF_1*α*_ in plasma rats. Each bar is the mean ± SD of concentration of circulating 6-keto-PGF_1*α*_ in each group. Differences between groups were determined by ANOVA procedure and S-N-K test. ^a^ *P* < 0.05 versus the control group, ^b^ *P* < 0.05 versus the SHR group.

**Figure 6:**
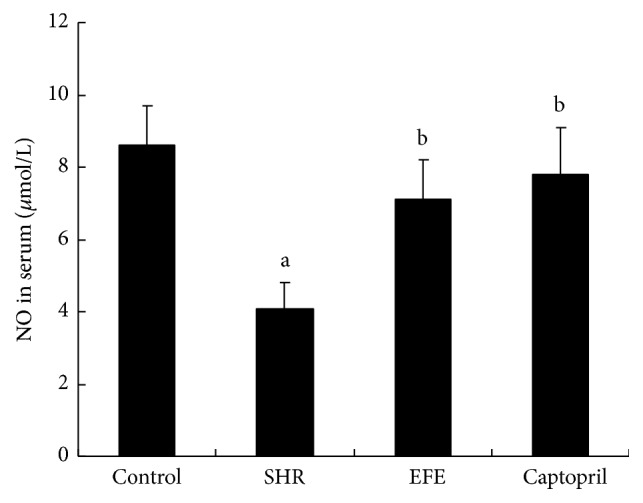
Concentrations of NO in serum of rats. Each bar is the mean ± SD of concentration of NO in each group. Differences between groups were determined by ANOVA procedure and S-N-K test. ^a^ *P* < 0.05 versus the control group, ^b^ *P* < 0.05 versus the SHR group.

NO and 6-keto-PGF_1*α*_ are substances that can decrease the peripheral vascular resistance, resulting in reduction in blood pressure. Serum NO concentration and plasma 6-keto-PGF_1*α*_ concentration were also measured after the 4 weeks of treatment; the results are, respectively, presented in Figures 5 and 6. In contrast to WKY rats, SHR rats in PBS group had markedly lower concentration of NO and 6-keto-PGF_1*α*_ (both *P* < 0.05). EFE and captopril treatments both significantly increased NO and 6-keto-PGF_1*α*_ levels in SHR rats if compared with the PBS treated SHR rats (all *P* > 0.05). Levels of NO and 6-keto-PGF_1*α*_ are comparable in EFE group, captopril group, and control group (all *P* > 0.05).

## 4. Discussion

The current study mainly demonstrates the fact that traditional Chinese medicine earthworm has ACE inhibitory active ingredient, and the extract (EFE) from earthworm has hypotensive effects in SHR rats.

As of 2000, nearly one billion people or about 26% of the adult population of the world had hypertension [30], which puts a large number of people at the risk of hypertensive heart disease, coronary artery disease, stroke, aneurysms of the arteries, peripheral arterial disease, and chronic kidney disease. Drug treatment is often necessary in people for whom lifestyle changes are not enough or not effective. Besides aiming at decreasing blood pressure, cardiovascular remodeling should be taken into account when using drugs. Among several classes of medications currently available for treating hypertension, ACEI is supposed to have beneficial effects on both blood pressure and cardiovascular remodeling [4, 5], which significantly lowers the risk of hypertensive complications and prolongs patients' lifetime.

Some biologically active compounds were recently isolated from various traditional herb drugs and have been shown to have ACE inhibitory activity [13–15]. Earthworm, which is also called Dilong in China, has long been used as a traditional medicine. It contains lots of compounds with potential medicinal properties and has been used to treat inflammation, arteriosclerosis [24], thrombus [31], heart diseases [25, 32], nerve disease [33], and asthma [34] in clinical and animal studies. However, less is known about the effects of earthworm and its extracts on blood pressure and RAS system. In the current study, we obtained an extract from* Eisenia fetida* by gel-filtration chromatography and determined its ACE inhibitory activity* in vitro*. The results showed this extract had an IC_50_ value of 2.5 mg/mL, which means it has a certain degree of ACE inhibitory activity. ACE is a dipeptide-liberating exopeptidase which can remove a dipeptide from C-terminus of angiotensin I to form angiotensin II.

In order to investigate whether the extract has effects in RAS system* in vivo*, we administrated it to SHR rats and determined the circulating Ang II levels by radioimmunoassay. Results showed SHR rats had markedly higher circulating Ang II levels than the WKY rats, while EFE treated SHR rats had markedly lower levels of Ang II than the PBS treated SHR rats. The results suggested that the extract also exerted inhibitory activity on RAS system* in vivo*. It is well known that Ang II is the most potent substance in RAS system and plays a crucial role in the pathophysiology hypertension. Management targeted at inhibition of Ang II production by ACEI has been shown to have various beneficial effects on hypertensive animals and patients [35, 36]. So we suppose that inhibition of Ang II production with the extract from earthworm should contribute to the recovery of hypertension. Besides the Ang II determination, we also measured circulating Ald levels of the rats by radioimmunoassay. Similarly, the 4-week treatment with the extract also decreased circulating Ald levels of SHR rats. Ald is also an important part of the RAS cascade reactions and contributes to the increase of blood pressure and cardiovascular remodeling [37, 38]. The decrease in Ald levels should be beneficial to the control of hypertension in this study.

Besides converting angiotensin I to angiotensin II, ACE also can inactivate bradykinin, which would block the bradykinin induced PGI2 and NO releases. Because PGI2 is an unstable metabolite of arachidonic acid, it is technically difficult to measure the accurate circulating PGI2 levels. As the stable hydrolysate of PGI2, 6-keto-PGF_1*α*_, accurately reflects the concentrations of its precursor, so we measured the circulating 6-keto-PGF_1*α*_ levels of the rats by radioimmunoassay. The result showed SHR rats treated with PBS had markedly lower circulating 6-keto-PGF_1*α*_ levels than the WKY rats, while EFE treated SHR rats had markedly higher levels of 6-keto-PGF_1*α*_ than the PBS treated SHR rats. The results suggested that the extract could increase PGI2 production* in vivo*. PGI2 is a prostaglandin member of the family of lipid molecules known as eicosanoids. Besides its action of vasodilation, it also has antiproliferative, antithrombotic, and anti-inflammatory effects. Management targeted to increase PGI2 production has been reported to be beneficial for hypertensive control [39]. So we speculate that, in the current study, the increased PGI2 production induced by the administration of the extract from earthworm should contribute to antihypertensive effects. Serum NO concentrations levels of the rats were also measured. Similar to the tendency of 6-keto-PGF_1*α*_, the extract from earthworm significantly evaluated serum NO concentrations of the SHR rats. Since NO is a very potent vasodilator, the evaluated NO should lead to the dilation of the blood vessels which would result in the decrease of blood pressure.

In addition to the detection of above vasoactive substances* in vivo*, we also directly observed the effects of the extract on SBP and DBP of the rats. Following the 4 weeks of treatment, SHR rats in SHR group and WKY rats in the control group treated with PBS showed no marked reduction of SBP and DBP if compared to their basal blood pressure; however, SHR rats in the EFE group and captopril group showed significant lower SBP and DBP than those of the SHR group and their respective basal levels; moreover, their SBP and DBP were similar to those of the WKY rats in the control group. The results suggested that the extract had a potent hypotensive effect in SHR rats.

We conclude that the extract from earthworm has ACE inhibitory activity* in vitro* and hypotensive effects* in vivo*. It can inhibit the levels of circulating Ang II and Ald, as well as promoting the 6-keto-PGF_1*α*_ and NO productions in SHR rats. Further study is needed to investigate beneficial effects of the extract on hypertensive damage of cardiovascular organs.
